# Quantitative Proteomics of Urinary Bladder Cancer Cell Lines Identify UAP1 as a Potential Therapeutic Target

**DOI:** 10.3390/genes11070763

**Published:** 2020-07-08

**Authors:** Vinuth N. Puttamallesh, Barnali Deb, Kirti Gondkar, Ankit Jain, Bipin Nair, Akhilesh Pandey, Aditi Chatterjee, Harsha Gowda, Prashant Kumar

**Affiliations:** 1 Institute of Bioinformatics, International Technology Park, Bangalore 560066, India; vinuth@ibioinformatics.org (V.N.P.); barnali@ibioinformatics.org (B.D.); kirti@ibioinformatics.org (K.G.); ankit@ibioinformatics.org (A.J.); pandey.akhilesh@mayo.edu (A.P.); aditi@ibioinformatics.org (A.C.); harsha@ibioinformatics.org (H.G.); 2 Amrita School of Biotechnology, Amrita Vishwa Vidyapeetham, Kollam 690525, India; bipin@am.amrita.edu; 3 Manipal Academy of Higher Education, Madhav Nagar, Manipal 576104, India; 4 Department of Laboratory Medicine and Pathology, Mayo Clinic, Rochester, MN 55905, USA; 5 Center for Individualized Medicine, Mayo Clinic, Rochester, MN 55905, USA; 6 Center for Molecular Medicine, National Institute of Mental Health and Neurosciences, Bangalore 560029, India

**Keywords:** quantitative proteomics, urothelial carcinoma, molecular subtypes, therapeutic target

## Abstract

Bladder carcinoma (BC) incidence and mortality rates are increasing worldwide. The development of novel therapeutic strategies is required to improve clinical management of this cancer. Aberrant protein expression may lead to cancer initiation and progression. Therefore, the identification of these potential protein targets and limiting their expression levels would provide alternative treatment options. In this study, we utilized a liquid-chromatography tandem mass spectrometry-based global proteomics approach to identify differentially expressed proteins in bladder cancer cell lines. A total of 3913 proteins were identified in this study, of which 479 proteins were overexpressed and 141 proteins were downregulated in 4 out of 6 BC cell lines when compared with normal human urothelial cell line (TERT-NHUC). We evaluated the role of UDP-N-acetylhexosamine pyrophosphorylase (UAP1) in bladder cancer pathogenesis. The silencing of UAP1 led to reduction in proliferation, invasion, colony formation and migration capability of bladder cancer cell lines. Thus, our study reveals UAP1 as a promising therapeutic target for bladder cancer.

## 1. Introduction

Bladder carcinoma (BC) is the most common malignancy of the urinary tract. Majority of the patients are diagnosed as non-muscle invasive (NMIBC; 60%) with a recurrence rate of 50%–70% and about 20% of patients are diagnosed as muscle invasive bladder cancer (MIBC) [1]. The risk of progression for NMIBC to MIBC after 5 years ranges from 6% to 45% [2,3]. MIBCs are biologically aggressive, with less than 15% five-year survival rate [4]. In the past few years, the surgical advancements have improved the outcomes of the patients. However, despite the use of neoadjuvant and adjuvant therapies, the long term survival rates in BC patients has remained unchanged for over a decade [5]. Furthermore, progress is required to develop a common molecular classifier that can be used for effective clinical decisions [6].

Several FDA approved immune checkpoint inhibitors, such as atezoliumab, nivoluma, pembrolizmab, durlumab and avelumab, were used to treat bladder cancer, with response rates ranging from 15% to 25% [7,8]. Moreover, extensive efforts have been made to identify therapeutic targets for BC, by targeting proteins belonging to various biological functions. This includes casein kinase 2 (CK2), C-X-C motif chemokine ligand 1(CXCL1), eukaryotic translation initiation factor 3 subunit D (EIF3D), adenylate kinase 4 and yes-associated protein 1 (YAP1) [9,10,11,12]. Alternatively, several proteins which are studied in other cancer types, such as are androgen receptor (AR), aurora kinase A (AURKA), epidermal growth factor receptor (EGFR), focal adhesion kinase (FAK), fibroblast growth factor receptor (FGFR) and transforming growth factor-β-induced (TGFBI) have also been explored in BC for their potential as therapeutic targets [13,14,15,16]. Though all these efforts provided multiple avenues for reducing the tumor burden in BC, they fell short in successful translation as treatment options.

Proteomics is a high-throughput technology that can be used to identify differentially expressed proteins that potentially play an important role in pathogenesis. A rapidly evolving technology platform is known to have the potential to identify novel proteins involved in key biological processes in the cell that may serve as potential drug targets. Therefore, an unbiased investigation of the proteomic alterations in BC using high resolution mass spectrometry-based approach will aid in identifying alternative therapeutic targets.

In this study, we investigated six BC cell lines (SW780, RT112, VMCUB1, T24, J82 and UMUC3), which were previously characterized as luminal, basal and non-type subtype [17]. Previously, it was shown that the EMT score of non-type molecular subtype is more “mesenchymal-like,” whereas the luminal/basal subtypes are “epithelial-like.” The non-type subtype cell lines show an increased migratory and invasive phenotype, reflecting typical characteristics of a mesenchymal-like phenotype [18]. We performed quantitative proteomic analysis to identify global proteomic changes in these BC cell lines. Our study identified and quantified 3913 proteins. We explored the role of UAP1 for the first time in bladder carcinoma, which was overexpressed in basal and non-type subtype cell lines. UAP1 plays an important role in energy metabolism and has been reported to be involved in prostate cancer pathogenesis [19]. Furthermore, the functional characterization of UAP1 using siRNA-based silencing in non-type subtype BC cell lines resulted in a decrease in the cell proliferation, colony formation, invasion and migration properties of highly aggressive bladder cancer cell lines.

## 2. Materials and Methods

### 2.1. Cell Lines and Culture Conditions

BC cell lines SW780, RT112, VMCUB1, T24, J82 and UMUC3 and normal human urothelial cell line TERT-NHUC (JTERT) were received from Prof. Jean Paul Thiery (Department of Biochemistry, National University of Singapore, Singapore). The non-type (UMUC3, J82, T24), luminal (RT-112, SW780) and basal (VMCUB-1) BC cell lines were cultured using DMEM medium supplemented with 10% Fetal Bovine Serum (FBS) and 1% Penicillin/Streptomycin. TERT-NHUC was grown in KGM gold^TM^ keratinocyte growth medium containing supplements (bovine pituitary extract, human epidermal growth factor, insulin, hydrocortisone, epinephrine, transferrin, gentamicin and amphotericin-B). All the cell lines were maintained at 37 °C in a humidified 5% CO_2_ incubator.

### 2.2. Cell lysis and Protein Digestion

Cells were lysed in lysis buffer (50 mM triethyl ammonium bicarbonate (TEABC) pH 8.0, 2% SDS, 1 mM sodium orthovanadate, 2.5 mM sodium pyrophosphate, 1 mM β-glycerophosphate, 1 mM sodium fluoride), sonicated and centrifuged at 16,000× *g* for 20 min. Protein estimation of the clarified lysate was done using bicinchoninic acid assay (BCA) (Pierce, Waltham, MA, USA) according to manufacturer’s instructions. Equal amount of protein from all the cell lines were reduced, using 10 mM Dithiotheritol (DTT) for 30 min at 60 °C, followed by alkylation using 20 mM Iodoacetamide (IAA) in dark for 10 min at room temperature. Reduced and alkylated protein lysate was subjected to acetone precipitation, using 7 volumes of pre-chilled acetone to remove SDS from the solution. Protein digestion was performed using sequencing grade trypsin (Promega, Madison, WI, USA) at 1:20 enzyme:substrateratio at 37 °C for 12–14 h. The tryptic peptides were vacuum dried and stored until further use.

### 2.3. TMT Labeling and Basic pH Reverse Phase Liquid Chromatography (bRPLC)

TMT labeling was done according to the manufacturer’s instructions, with minor modifications. Briefly, the TMT labels were reconstituted in 41µL of anhydrous acetonitrile (ACN) and trypsin digested peptide samples were reconstituted in 100 µL of 50 mM TEABC (pH 8.0). Tandem mass tag (TMT) labels 128N, 128C, 129C, 130N, 130C and 131 were used for labeling bladder cancer cell line samples, and TMT label 126 was used for TERT-NHUC control cell line. TMT labels were mixed with respective samples and the reaction was incubated for 1 h at room temperature. After incubation, the reaction was quenched with 8 µL of 5% hydroxylamine. The labeled peptides were lyophilized and subjected to basic pH reverse phase chromatography (bRPLC). Lyophilized samples were reconstituted in bRPLC solvent A (10 mM TAEBC, pH 9) and were separated on XBridge BEH C18 Column (Waters, UK), using solvent B (10 mM TEABC with 90% acetonitrile, pH 9). The column was equilibrated at 5% Solvent A from 0-to-5 min; the solvent B percentage was gradually increased from 5% to 55% in the following 60 min and then increased from 55–90% for the following 10 min, and then maintained at 90% Solvent B for 10 min before being decreased to 5% for 2 min on an Agilent 1100 Liquid Chromatography (LC) system, with a flow rate of 1 mL/min. A total of 96 fractions were collected over a period of 60 min gradient and later concatenated into 12 fractions. Fractionated samples were lyophilized before LC-MS/MS analysis.

### 2.4. LC-MS/MS Analysis

LC-MS/MS analysis was done on an Orbitrap Fusion mass spectrometer (Thermo Electron, Bremen, Germany), interfaced with Easy-nLC1000 nanoflow LC system (Thermo Scientific, Odense, Denmark). The peptides were reconstituted in 0.1% formic acid and loaded onto a trap column (nanoviper 2 cm, 3 μ magic C18Aq, Thermo Scientific). Peptides were resolved on an analytical column (nanoviper 25 cm (75 µm silica capillary, 3 µm magic C18, Thermo Scientific)), at a flow rate of 300 nL/min, using a linear gradient of 7–35% solvent B (0.1% formic acid in 100% ACN) over 100 min. The total run time including sample loading and column reconditioning was 120 min. Data-dependent acquisition with full scans in 350–1700 m/z range was carried out using an Orbitrap mass analyzer at a mass resolution of 120,000 at 400 m/z. Most intense precursor ions from a survey scan were selected for MS/MS fragmentation using higher energy collision dissociation (HCD) fragmentation, with 35% normalized collision energy and detected at a mass resolution of 30,000 at 400 m/z. AGC target value was set to 50,000, with maximum ion injection time of 150 ms. For MS3 analysis, synchronous precursor selection was enabled and 10 precursor ions were selected for fragmentation with 55% HCD collision energy.

### 2.5. Data Analysis

The mass spectrometry data were searched for using MASCOT (version 2.2.0) and SEQUEST search algorithms against Human RefSeq database (version 89), using Proteome Discoverer (version 2.2 (Thermo Fisher Scientific, Bremen, Germany). The search parameters for both algorithms included carbamidomethylation of cysteine residues (57.02 Da), TMT modification at peptide N-terminus and lysine side chain as a fixed modification, oxidation of methionine (15.99 Da) as dynamic modification. MS/MS spectra were searched with a precursor mass tolerance of 10 ppm and fragment mass tolerance of 0.1 Da. Trypsin was specified as the protease, and a maximum of two missed cleavages were allowed. The data was searched against target decoy database and the false discovery rate was set to 1% at the peptide level. The TMT ratio for each peptide-spectrum match was calculated by the quantitation node.

### 2.6. Protein-Protein Interaction Networks

Interaction network was analyzed using the STRING functional protein association network (https://string-db.org; version: 11.0; University of Zurich, Zurich, Switzerland) [20]. The input consisted of the 122 proteins that were overexpressed in all the BC cell lines and was set to highest confidence (0.90) of active interaction. The disconnected nodes were hidden, and K-means clustering was conducted to identify three clusters in the dataset.

### 2.7. Western Blotting

Cell lines were cultured up to 70% confluency and cells were harvested using RIPA lysis buffer (10 mM Tris pH 7.4, 150 mMNaCl, 5mM EDTA, 1% Triton-X-100, 0.1% SDS containing protease and phosphatase inhibitor cocktails) and sonicated to extract proteins. Western blot analysis was performed as previously described, using 30 µg protein lysates [21]. Nitrocellulose membranes were hybridized with primary antibodies and developed using Luminol reagent (Santa Cruz Biotechnology, Dallas, TX, USA), as per the manufacturer’s instructions. Anti-UAP1 (HPA014659) antibody and β-actin antibody were obtained from Sigma (St. Louis, MO, USA). Anti-GAPDH antibody was obtained from Abcam (Cambridge, MA, USA).

### 2.8. siRNA Transfection

ON-TARGETplusSMARTpool control siRNA and UAP1siRNA (catalog ID: L-017160-01-0005) were procured from Dharmacon (Lafayette, CO, USA), and BC cell lines (UMUC3, J82 and T24) were transfected with controlandUAP1siRNA using RNAiMAX reagent (Invitrogen, Carlsbad, CA, USA), according to the manufacturer’s instructions. Cells were subjected to invasion assays and colony formation assays 48 h post-transfection, unless otherwise stated.

### 2.9. Cell Proliferation Assay

Cell proliferation assays were carried out as described previously [22]. Cells from UMUC3, J82 and T24 cell lines were seeded at a density of 4000 cells/well into a 96-well plate and cells were counted subsequently after every 48 h. Cell proliferation was determined using MTT (3-(4, 5-dimethylthiazol-2yl)-2, 5-diphenyl tetrazolium bromide) assays, as described. Absorbance was measured at 570 nm and 650 nm over a period of 4 days and growth kinetics were plotted. All the experiments were carried out in triplicates.

### 2.10. Colony Formation Assay

Colony formation assays were carried out as described previously, with minor modifications [21]. UMUC3, J82 and T24 cells were transfected with UAP1siRNA or scramble siRNA. 48 h post transfection, 1000 cells/well were seeded in 6-well plates with complete media and allowed to grow for 7 days. The resulting colonies were fixed with methanol, and stained with Giemsa (Sigma, St. Louis, MO, USA). The number of colonies per dish was counted. All experiments were performed in triplicate and standard deviation was calculated.

### 2.11. Wound Healing Assay

The wound healing assays were performed as described previously [23]. Briefly, 100,000 cells were seeded in 6 well plates in triplicates for each condition. UMUC3, J82 and T24 cells were treated with UAP1siRNA or control siRNA. The cells were allowed to grow until full confluency was achieved. Uniform size wound was introduced from end to end in a 6 well plate for each condition and a photograph was taken at 0 h. Cells were then observed for wound healing periodically and photomicrograph at 8 h was taken under microscope. All experiments were performed in triplicate, unless otherwise indicated.

### 2.12. Invasion Assay

Invasion assays were performed in a transwell system (BD Biosciences), with Matrigel-coated filters and cellular invasion was evaluated after 48 h, as described previously [24]. Briefly, invasiveness of the cells was assayed in the membrane invasion culture system using polyethylene terephthalate (PET) membrane (8-μm pore size), in the upper compartment of a transwell coated with Matrigel (BioCoat Matrigel Invasion Chamber; BD Biosciences). The cells were transfected with either control or UAP1siRNA and seeded at 2.0 × 10^4^ cells per 500 μL of media on the Matrigel-coated PET membrane in the upper compartment. The lower compartment was filled with complete growth media and the plates were maintained at 37 °C for 48 h. At the end of the incubation time, the upper surface of the membrane was wiped with a cotton-tip applicator, to remove non migratory cells. Cells that migrated to the lower side of membrane were fixed and stained using 4% methylene blue. The membranes were cut out from the transwell and mounted on glass slide using Dibutylphthalate Polystyrene Xylene (DPX) and covered with a microscope cover slip. The number of cells that penetrated was counted for six randomly selected viewing fields at 10x magnification.

### 2.13. Statistical Analysis

Statistical analyses were carried out using GraphPad Prism version 6 (GraphPad Software, La Jolla, CA, USA). For cell culture-based assays (proliferation, invasion, colony formation and wound healing) non-parametric test (Mann–Whitney U test) was used to assess statistical significance. For proteomics data, a statistical analysis was done using one-way ANOVA for individual proteins.

## 3. Results

### 3.1. Identification of Proteins with Altered Expression in Urinary Bladder Cancer Cell Lines through Quantitative Proteomics

**Figure 1:**
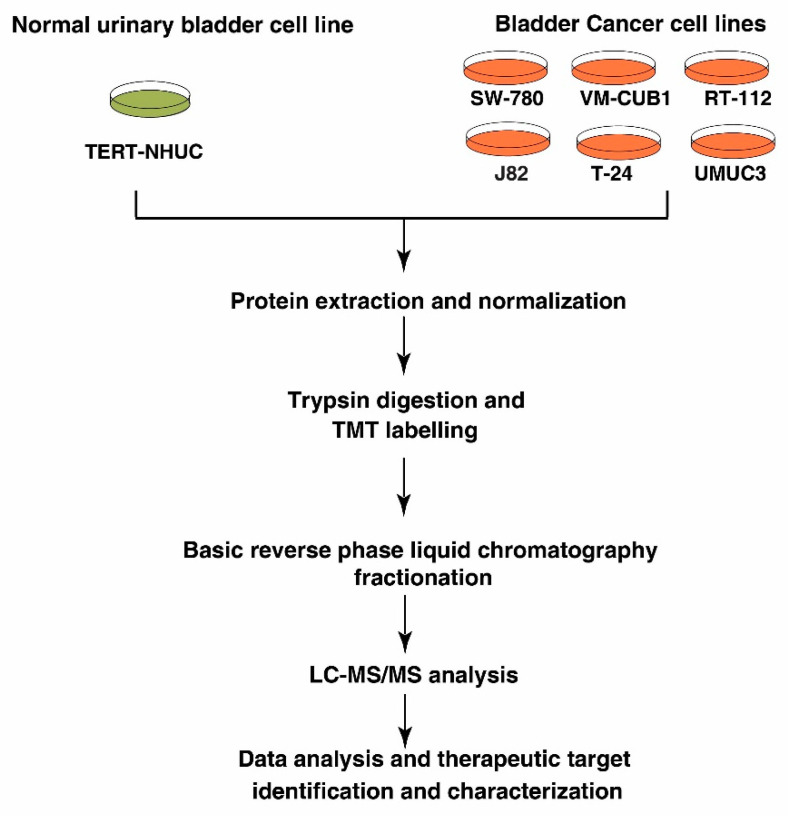
Workflow for quantitative proteomic analysis of urinary bladder cancer cell lines. Cultured bladder cancer (BC) cell lines and normal human urothelial carcinoma cell line (TERT-NHUC) were harvested in cell lysis buffer. Protein extraction was done using probe sonicator, followed by protein estimation, normalization, trypsin digestion and tandem mass tag (TMT) labeling. TMT labeled peptides were pooled and subjected to fractionation and LC-MS/MS analysis. Proteomics data were analyzed and candidate molecule was validated.

We studied the protein expression of BC cell lines (SW780, RT112, VMCUB1, T24, J82 and UMUC3) and normal human urothelial carcinoma cell line (TERT-NHUC) using tandem mass tag (TMT)-based labeling technology, coupled with high-resolution mass spectrometry to identify differentially expressed proteins. The experimental workflow used for differential proteomic expression analysis in this study is depicted in Figure 1. We identified and quantified 3913 proteins across all the cell lines in triplicate mass spectrometry analysis and calculated their fold-change values based on reporter ion intensities (Supplementary Table S1; Supplementary Figure S1).

### 3.2. Distinct Protein Expression Pattern of Non-Type/Basal as Compared to Luminal Bladder Carcinoma Cell Lines

**Figure 2:**
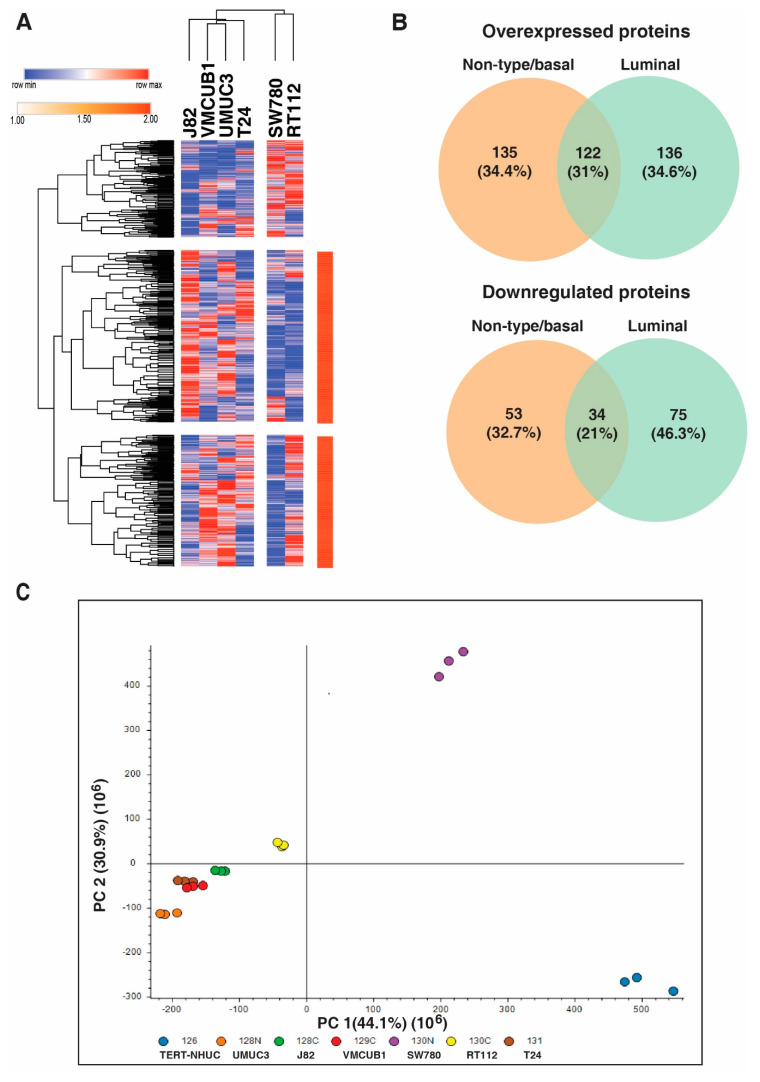
Comparison of the protein expression of non-type/basal and luminal subtype. (**A**) Unsupervised clustering showing the distinct expression pattern of non-type/basal and luminal cell lines. (**B**) Overexpressed and downregulated proteins in non-type/basal and luminal cell lines. (**C**) Principle component analysis of the protein expression of the bladder carcinoma cell lines.

We compared the protein expression pattern of the non-type/basal and luminal subtypes of bladder carcinoma cell lines. Unsupervised clustering based on both rows and columns was conducted with one minus Kendall’s correlation with average linkage and we observed that the luminal cell lines showed a distinct expression pattern (Figure 2A). We compared differentially expressed proteins (>2 fold) from all the non-type/basal and luminal cell lines. We identified 135 and 53 proteins, which were overexpressed and downregulated exclusively in non-type/basal cell lines (Figure 2B). Principle component analysis revealed that the luminal cell lines show distinct expression pattern as compared to the non-type/basal cell lines (Figure 2C).

### 3.3. DNA Replication Process and Cell Cycle Regulation and Progression Were Dysregulated across All Bladder Carcinoma Cell Lines

**Figure 3:**
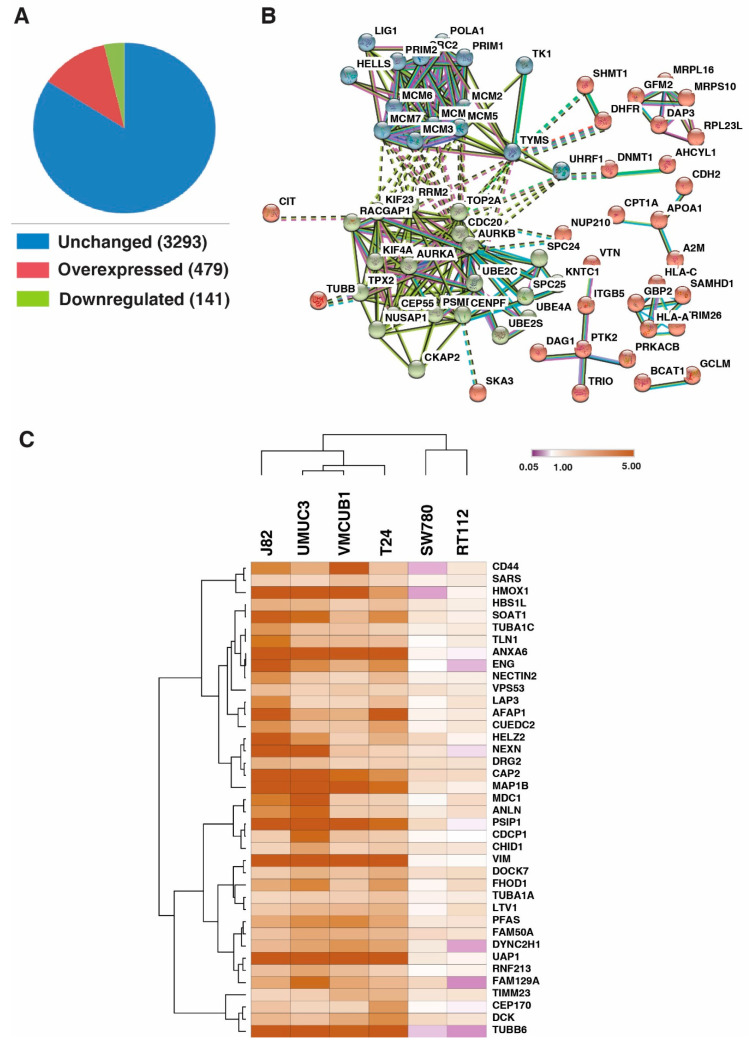
Global proteomic profiling of bladder carcinoma cell lines. (**A**) Total dysregulated proteins identified in the study in four or more bladder carcinoma cell lines, with 479 proteins overexpressed and 141 proteins downregulated. (**B**) Protein-protein interaction network showing the clusters of proteins with highest confidence (0.90) of interaction. (**C**) Heatmap showing 39 proteins that were overexpressed in the basal and non-type bladder carcinoma cell lines.

We identified 122 proteins to be overexpressed and 34 proteins to be downregulated across all the 6 BC cell lines, compared to the TERT-NHUC (control) cell line (Figure 3A). 479 proteins were overexpressed, and 141 proteins were downregulated ≥2 fold in four or more BC cell lines when compared with the TERT-NHUC (control) cell line. We checked the interaction between the proteins that were overexpressed across all BC cell lines. We observed that the proteins formed two major clusters; one cluster involved the MCM proteins; namely MCM2, MCM3, MCM4, MCM5, MCM6 and MCM7, which closely interact with each other and are involved in DNA replication, while the other cluster involved proteins involved in cell cycle regulation and progression, such as AURKB, AURKA, CDC20, RRM2, TOP2A, RACGAP KIF23, KIF4A, TPX2, CEP55, NUSAP1, CKAP2, CENPF, and so on (Figure 3B).

### 3.4. UAP1 Was Overexpressed in Bladder Carcinoma

We sought to identify the proteins that show higher expression in the basal and non-type subtype of bladder carcinoma, which might be attributed to the aggressive phenotype in these cells. We identified 39 proteins which were overexpressed in non-type and basal subtype (Figure 3C; Supplementary Table S2). We selected UDP-N-acetylhexosamine pyrophosphorylase (UAP1) as a candidate molecule to study the functional implication in BC, based on its function and association with cancer pathogenesis. Expression pattern of UAP1 in BC cell lines is represented in the box plot, as shown in Supplementary Figure S2.

### 3.5. Silencing of UAP1 Decreases Cellular Proliferation in Urinary Bladder Cancer Cells

**Figure 4:**
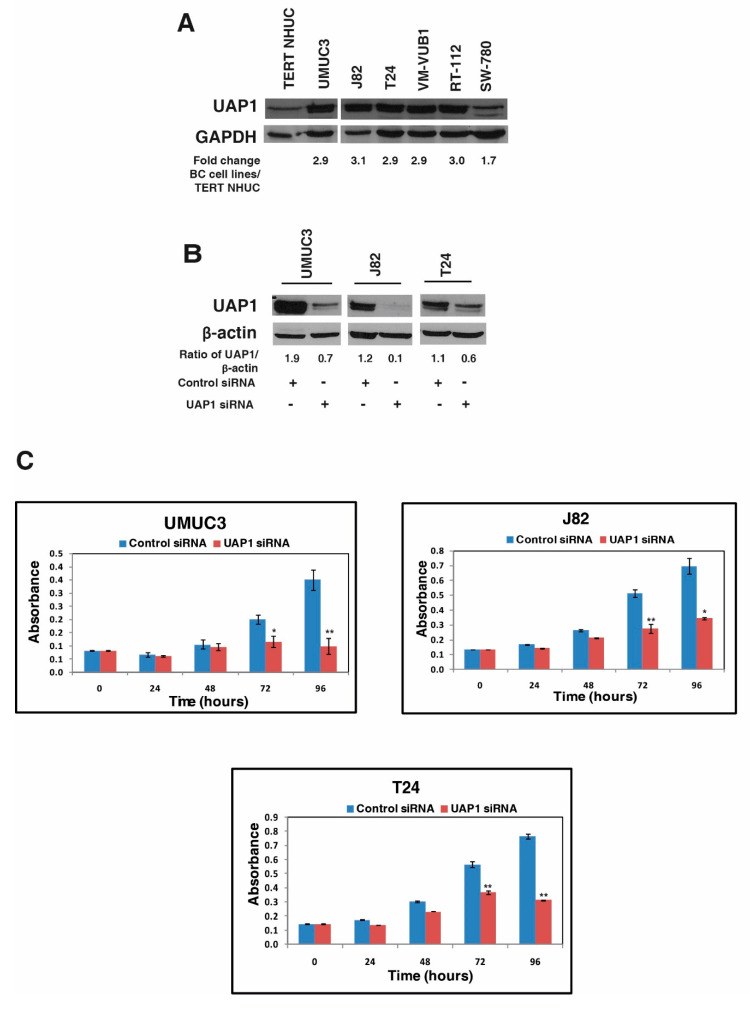
UAP1 silencing reduces cell proliferation of urinary bladder cancer cells. (**A**) Western blot analysis of UAP1 expression in BC cell lines and TERT-NHUC cell line validates the high expression of UAP1 in BC cell lines, as discovered from mass spectrometry analysis. (**B**) Western blot analysis of UAP1 silencing using siRNA mediated knockdown confirms the reduced expression of UAP1 after silencing in BC cell lines; β actin was used as a loading control. (**C**) Inhibition of UAP1 reduces cellular proliferation of BC cell lines (* *p* < 0.05; ** *p* < 0.01).

Western blotting analysis confirmed the overexpression of UAP1 in most of the BC cell lines compared with the TERT-NHUC cell line (Figure 4A). We selected the 3 non-type BC cell lines (UMUC3, J82, T24), to study the role of UAP1 in cellular proliferation. Endogenous expression of UAP1 was silenced-using siRNA mediated knockdown and Western blot analysis confirmed the successful knockdown of UAP1 in BC cell lines (Figure 4B). We then assessed the effect of UAP1 knockdown on the cellular proliferation of BC cell lines. UAP1 siRNA and control siRNA transfected BC cell lines were grown in triplicate for 96 h, and cell viability was assessed at every 24 h intervals using MTT assay. We observed significant reduction in cellular proliferation of UAP1 knockdown BC cell lines when compared with the control cell line (Figure 4C). These results indicate that UAP1 plays a role in cellular proliferation in non-type BC cells.

### 3.6. Silencing of UAP1 Decreases Colony Forming Ability in Urinary Bladder Cancer Cells

**Figure 5:**
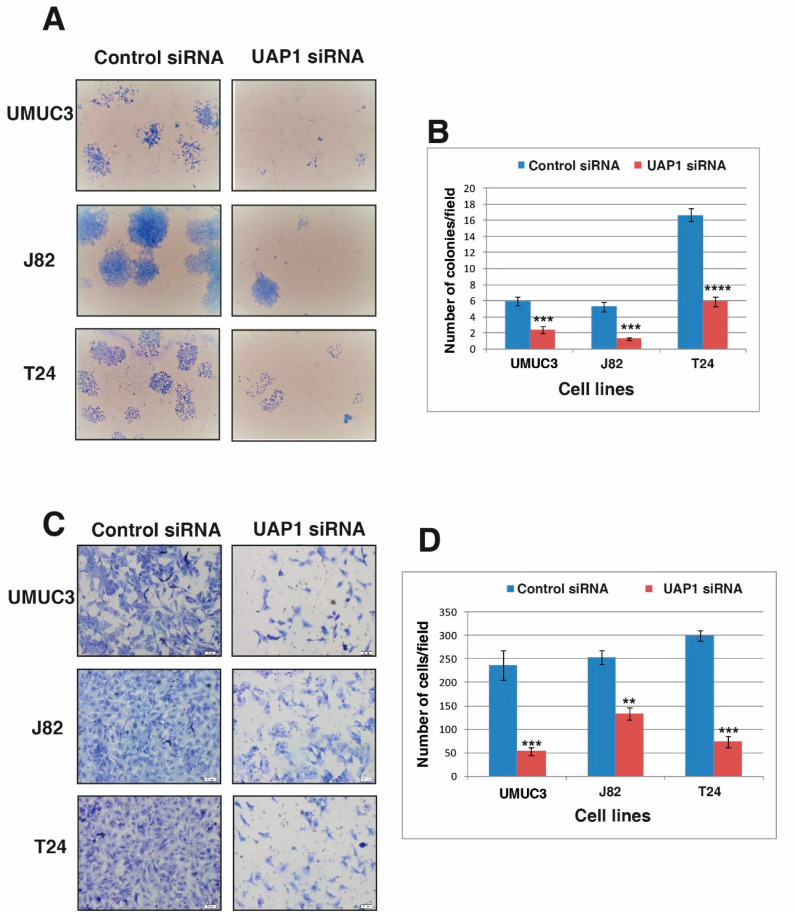
Inhibition of UAP1 affects the colony forming ability and reduces the invasive ability of urinary bladder cancer cell lines. (**A**) Colony formation assay was performed after siRNA mediated knockdown of UAP1 or control siRNA in BC cell lines. Colonies formed were fixed and stained using methylene blue. Images were captured and colonies were counted; reduced colony forming ability of BC cells after siRNA silencing was observed. (**B**) Graphical representation of reduction in colony forming ability of BC cell lines after UAP1 silencing (*** *p* < 0.001; **** *p* < 0.0001). (**C**) BC cell lines were transfected with UAP1 siRNA or control siRNA and invasion assay was performed. The assay was done in transwell system using Matrigel-coated filters and the cells that migrated to the lower chamber fixed, stained and images captured for counting and representation. (**D**) Graphical representation of reduction in invasive ability of BC cell lines after UAP1 silencing (** *p* < 0.001; *** *p* < 0.001).

After demonstrating the role of UAP1 in cellular proliferation, we continued to study the role of UAP1 in the colony forming ability of the BC cells. UAP1 siRNA transfected cells and control siRNA transfected cells were seeded onto 6 well plates at 1000 cells per well density and allowed to grow for 7 days. The colonies formed were fixed, stained, counted under microscope and photographed for representation. Silencing of UAP1 led to reduction in the number of colonies formed and reduced the size of colonies formed in UAP1 knockdown non-type BC cells (Figure 5A,B). Our results show the importance of UAP1 in the colony formation of BC cells.

### 3.7. Silencing of UAP1 Decreases Invasive Property in Urinary Bladder Cancer Cells

We further examined whether UAP1 has a role in BC invasiveness. Invasion assays were performed after silencing the endogenous expression of UAP1 using siRNA. BC cells transfected with UAP1 siRNA or control siRNA were transferred to the matrigel-coated PET membrane in the upper compartment and the plates were incubated at 37 °C for 48 h. We fixed and stained the migrated cells and counted the cells under a microscope. siRNA mediated silencing of UAP1 showed reduction in invasive capability of all the non-type BC cell lines (Figure 5C,D). Our results indicate a clear role of UAP1 in regulating invasive capability, which is a critical process during metastasis.

### 3.8. Silencing of UAP1 Decreases Cell Motility in Urinary Bladder Cancer Cells

**Figure 6:**
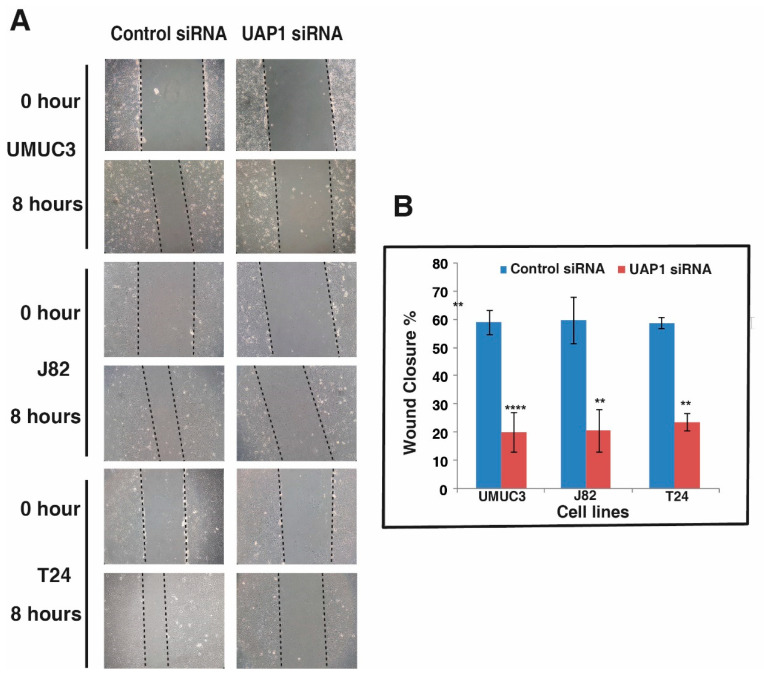
Inhibition of UAP1 reduces the migratory ability of bladder cancer cell lines. (**A**) Wound healing assay was carried out after transfection of BC cell lines using either UAP1 siRNA or control siRNA; scratches were made after cell confluence and monitored for 8 h till wound healing. (**B**) Representative graph shows the distance migrated by BC cell lines (* *p* < 0.05; ** *p* < 0.01; *** *p* < 0.001; **** *p* < 0.0001).

Since the silencing of UAP1 reduces both the cell proliferation and colony forming ability of non-type subtype BC cells, we decided to explore whether UAP1 has any role in tumor cell motility. We performed scratch wound assays using non-type subtype BC cells with or without UAP1 silencing. Cells were grown till full confluence in 6 well plates and wounds were made in uniform size. After incubation for 8 h, control BC cells showed increased migration compared to UAP1 silenced BC cells. We took the images of wounds at 0 h and 8 h and calculated the distance covered by cells using Image J software I (Figure 6A,B). Our results from the cell motility assay clearly showed that UAP1 influences cell motility in BC cells.

## 4. Discussion

BC management is challenging due to its presentation, histological subtypes and high recurrence rates. Surgical intervention and systemic chemotherapy are the preferred treatment options by clinicians, due to lack of bladder cancer specific targeted therapies [25]. The deeper knowledge on molecular mechanisms would help enormously to develop precision therapeutics. In addition, alternative therapeutic approaches need to be explored to improve the overall survival of patients, particularly in the case of aggressive tumors. In this study, we have conducted a global quantitative proteomics study to identify dysregulated proteins and potential targets which could lead to better treatment strategies in bladder carcinoma.

Cancer cell lines provide abundant information about cancer cell biology and alterations in protein repertoire in disease pathogenesis. In this study, we identified 122 proteins that were overexpressed across all the BC cell lines. These proteins might play an important role in cancer pathogenesis. The protein-protein interaction network revealed interaction between MCM proteins which formed the hub proteins. These proteins include a panel of minichromosome maintenance complex component (MCM) proteins; namely MCM2, MCM3, MCM4, MCM5, MCM6 and MCM7 [26,27]. MCM complex plays an important role in the DNA replication initiation. The active MCM2-7 double hexamer conducts bidirectional DNA synthesis at the S-phase of the cell cycle [28,29]. It has been reported that MCM complex subunits have been implicated in cell proliferation, invasion and metastasis in cancer [30,31,32]. A meta-analysis of the MCM proteins also reported that the higher expression of these proteins were related to worse prognosis in cancer patients [28]. Indeed, MCM expression is often observed in all epithelial layers and a high frequency of MCM-positive cells correlates with adverse clinical outcome in bladder carcinoma [33].

Apart from MCM family proteins, we also identified thymidine kinase (TK1), aurora kinase B (AURKB), cell division cycle protein 20 (CDC20), DNA topoisomerase 2-α (TOP2A), exportin-5 (XPO5) and several other proteins significantly overexpressed in our study [34,35,36,37]. Pharmaceutical compounds targeting Aurora A (such as Alisertib), have been extensively studied in preclinical models and observed to have synergy with several other targeted therapies that lead to tumor regression in various cancers [38]. Furthermore, TK1 has been reported as a tumor biomarker and it also exhibits potential in drug discovery and as a therapeutic target [39]. Interestingly, TK1 is not essential for normal cell growth, but it is important in the repair of DNA damage that may be caused by chemotherapy [6]. Alternatively, the overexpression of TK1 can reduce the efficacy of chemotherapy as well [39]. Cell cycle proteins are being widely studied for their potential in cancer therapeutics and further mechanistic studies would lead to their clinical utility in personalized therapy.

Our proteomics data identified 39 proteins which were specifically overexpressed more than 2-fold in basal and non-type subtype cell lines compared to the luminal subtype (table A). Among the list, only few molecules have been previously studied in bladder cancer tumorigenesis. Annexin-6 was overexpressed in pT1 grade3 in urothelial carcinoma [40]. Expression of vimentin was found in 69% cases of transitional cell carcinoma and its expression is associated with the grade of transitional cell carcinoma [41]. In accordance with our study, Wu et al. have also identified the expression of Anilin actin binding protein to be subtype-specific [42]. Similarly, the expression of Anilin actin binding protein was higher in the basal subtype compared to luminal subtype in bladder cancer cell lines.

Aberrant glycosylation has been gaining importance as one of the hallmarks of cancer. Alteration in metabolism and dysregulation in cellular energetics leads to cancer progression [43]. A tight association between the hexosamine biosynthetic pathway (HBP) and cell metabolism is widely reported [44]. Post-translational modification like glycosylation plays a key role in cell adhesion, migration, immune surveillance, cell signaling and cellular metabolism. HBP mediates glycosylation events through the final substrate UDP-GlcNAc. The conversion of GlcNAc-1P substrate to UDP-GlcNAc is mediated by UDP-N-acetylhexosamine pyrophosphorylase (UAP1) [45].

In our data, we identified that UDP-N-acetylhexosamine pyrophosphorylase (UAP1) was overexpressed more than 2-fold in all the basal and non-type subtypes of bladder cancer cell lines. Although the lack of biological replicates for proteomic analysis of each cell line is a limitation, UAP1 was chosen for further characterization, as it was overexpressed in four out of six cell lines analyzed. Functional assays to evaluate role of UAP1 in regulating proliferation, cell migration and colony formation were carried out in biological replicates across three different cell lines. UAP1 is the final enzyme in the hexosamine biosynthetic pathway (HBP), producing UDP-N-Acetylglucosamine (UDP-GlcNAc), which is a substrate for protein glycosylation. Abnormal protein glycosylation was previously shown to be associated with poor prognosis in bladder cancer patients [45]. A previous study in prostate cancer has shown the ability of UAP1 to protect cancer cells from endoplasmic reticulum stress and provide a growth advantage. High expression of UAP1 exhibited resistance against inhibitors of N-linked glycosylation (tunicamycin and 2-deoxyglucose) and targeting UAP1 blocked anchorage independent growth [19]. Earlier work from our group on N-glycoproteomic profile in bladder cancer cell lines has identified aberrant glycosylation in aggressive non-type subtype BC cell lines [46]. These results prompted us to further investigate UAP1 role in BC. Western blot analysis in our study confirmed that UAP1 was also overexpressed in the luminal cell lines, although not as high as in the basal or non-type cell lines. Further studies are required to understand its role in cancer progression, to comment on its role on the aggressive phenotype. Furthermore, knockdown of UAP1 using siRNA-based silencing in vitro showed a reduction in cell proliferation, invasion, colony formation and migration properties of BC cell lines. These results are novel to BC and demonstrate UAP1 role in tumorigenesis.

## 5. Conclusions

In conclusion, comprehensive quantitative proteomic analysis of urinary BC cell lines identified the overexpression of UAP1 for the first time. Knockdown of UAP1 reduced BC cell proliferation, invasion, colony formation and migration ability of these cell lines. Our data suggests that UAP1 could be a promising therapeutic target for aggressive urinary bladder cancer.

## Supplementary Materials

The following are available online at https://www.mdpi.com/2073-4425/11/7/763/s1, Figure S1: Unsupervized clustering of all the proteins identified and quantified across 6 BC cell lines, Figure S2: Box plot representing expression pattern of UAP1 in BC cell lines. Table S1: Complete list of proteins identified and quantified in the study, Table S2: Proteins overexpressed in non-type and basal subtype BC cell lines.
